# Virtual Screening of Natural Compounds as Potential PI_3_K-AKT1 Signaling Pathway Inhibitors and Experimental Validation

**DOI:** 10.3390/molecules26020492

**Published:** 2021-01-18

**Authors:** Serena Dotolo, Carmen Cervellera, Maria Russo, Gian Luigi Russo, Angelo Facchiano

**Affiliations:** Institute of Food Science, Italian National Research Council (ISA-CNR), via Roma 64, 83100 Avellino, Italy; sdotolo@unisa.it (S.D.); carmen.cervellera@isa.cnr.it (C.C.); maria.russo@isa.cnr.it (M.R.); glrusso@isa.cnr.it (G.L.R.)

**Keywords:** AKT1, pharmacophore model, virtual screening, molecular docking, ADMET analysis, kinase inhibitors

## Abstract

A computational screening for natural compounds suitable to bind the AKT protein has been performed after the generation of a pharmacophore model based on the experimental structure of AKT1 complexed with IQO, a well-known inhibitor. The compounds resulted as being most suitable from the screening have been further investigated by molecular docking, ADMET (Absorption, Distribution, Metabolism, Excretion, and Toxicity) analysis and toxicity profiles. Two compounds selected at the end of the computational analysis, i.e., ZINC2429155 (also named STL1) and ZINC1447881 (also named AC1), have been tested in an experimental assay, together with IQO as a positive control and quercetin as a negative control. Only STL1 clearly inhibited AKT activation negatively modulating the PI_3_K/AKT pathway.

## 1. Introduction

Protein kinase B/(PKB)/AKT belongs to the ACG family of serine-threonine kinases. It has been identified about 30 years ago by the cloning of the v-AKT oncogene from the transforming murine retrovirus AKT-8 [1]. Four years later, different laboratories identified the cellular homolog of v-AKT, an approximately 57 kDa protein kinase called c-AKT [2] or PKB, for its similarity with PKA/PKC kinases [3,4]. In the 90s, PKB/AKT acquired a prominent role in the field of cellular signal transduction, when it became clear that its activation was downstream of the lipid kinase PI_3_K (phoshoInositide-3-kinase), closely related to tumor transformation and cellular response to insulin [5]. Currently, PKB/AKT is well known to regulate key cellular functions such as metabolism, survival and proliferation in response to hormones and growth factors. This evidence explains why the over-activation of PKB/AKT caused by excessive production of the membrane lipid PIP_3_ (phosphatidyl-Inositol-3,4,5-tris-phosphate), produced by PI_3_K, is observed in 50% of human cancers [6,7,8], while defects in the PKB/AKT pathway is associated with metabolic diseases (diabetes and insulin resistance) [9]. Three AKT isoforms have been characterized. They show high sequence identity (>80%) and are named AKT1 (PKBα), AKT2 (PKBβ) and AKT3 (PKBγ), encoded by three distinct genes located on different chromosomes. These isoforms share a common architecture consisting of a catalytic domain flanked by an N-terminal domain called PH (Pleckstrin Homology) and a C-terminal regulatory domain (EXT) characterized by a hydrophobic motif (HM), typical of ACG kinases [10]. In vivo mutagenesis studies (knockout mice) demonstrated that each isoenzyme possesses a typical function: in particular, mice with the specific AKT1 deletion are characterized by growth delays and perinatal lethality. AKT2 knock-out mice develop insulin resistance and a type II diabetes-like syndrome, while mice deleted for AKT3 show a decrease in the brain volume [11]. PH domain is highly conserved in the different AKT isoforms (sequence homology between 76 and 84%) for its essential role in the interaction with membrane phospholipids such as PIP_3_ produced by PI_3_K. Biochemical analysis revealed that the PH domain of PKB/AKT binds with similar affinity to both PIP_3_ and PIP_2_ (phosphatidyl-inositol-3,4,-di-phosphate) [12]. The binding of phosphoinositides to the PH domain causes conformational changes allowing the phosphorylation of the Thr308 and Ser473 residues by PDK1 and mTORC2 (mechanistic target of rapamycin (mTOR) complex 2) kinases, respectively, leading to the full AKT enzymatic activation [13]. Phosphorylation in Thr408 residue in the C-terminal regulatory domain called turn motive (TM) stabilizes the AKT protein in the cytosol after its synthesis. Signal termination is instead controlled by PIP3-dependent phosphatase (PTEN) and by phosphatases PP2A and PHLPP [13].

Activated PKB/AKT can phosphorylate over 100 bona fide substrates where the targets Ser/Thr residues are embedded in the minimal consensus motif: R-X-R-X-X-S/T-ϕ (where X stays for any amino acid and ϕ refers to a large hydrophobic residue). However, important substrates, such as AMP-regulated protein kinase (AMPK) have in the PKB/AKT consensus phosphorylation site a Pro residue at the −5 position, rather than the typical R (Arg) residue [14]. Important physiological substrates of PKB/AKT are GSK3β (glycogen synthase kinase β), which is inactivated by phosphorylation in Ser9 resulting in a final proliferative and survival response; the transcription factor FoxO, which, upon phosphorylation, translocates to the cytoplasm blocking de facto the transcription of genes that arrest the cell cycle and/or induce apoptosis [15].

Due to its peculiar role as a central “cellular hub” in proliferation, growth and metabolism, the PI_3_K/AKT axis is one of the most frequently altered biochemical pathways in the complex network of intracellular signals leading to human cancers. These alterations include somatic oncogenic amplifications or mutations impacting the regulation or the expression of proteins such as EGFR, HER2, PKD1 and PIK3CA receptors [13]. For these reasons, the PI_3_K/AKT pathway became a key target for the latest generation of anticancer drugs, characterized by high molecular specificity to minimize the cytotoxic side effects on normal cells [11]. These innovative drugs can target a specific member within the pathway (e.g., PI_3_K, AKT and mTOR) and have been or are being tested in clinical trials. This is the case, for example, of Idelalisib (CAL-101), the first PI_3_K inhibitor approved by the US Food and Drug Administration (FDA), which targets the isoform δ of the catalytic subunit of PI_3_K (p110 δ) [16]. Idelalisib is effective against chronic lymphocytic leukemia (CLL), follicular lymphoma (FL) and small lymphocytic lymphoma (SLL) [17]. Several synthetic compounds that directly target AKT and showed promising results in animal models of cancer reached phase I/II clinical trials. They have been functionally characterized, but the results are still debatable since the complexity of the AKT signaling pathway network generates phenomena of compensatory resistance reactivating PI_3_K or mTOR in the PI_3_K/AKT/mTOR pathway. Therefore, the combination of inhibitors that hit more than one kinase in the PI_3_K/AKT/mTOR phosphorylation cascade appears as a promising therapeutic alternative against different types of cancer [7].

The possibility that natural agents can inhibit the PI_3_K/AKT pathway gained great interest and the efficacy of several phytochemicals were analyzed in recent review articles [7,18]. A good example is quercetin that, in cellular models of CLL, and in association with the BH3-mimetic ABT-737, synergistically enhanced apoptosis by directly targeting PI_3_K and CK2 kinases, being the latter a positive modulator of the PI_3_K/AKT pathway since it inactivates PTEN phosphatase [19,20]. However, only in a few cases, a direct interaction between the natural agent and AKT has been demonstrated. As an example, [6]-Shogaol from ginger root can directly target AKT1 and AKT2, but not PI_3_K or mTOR, suppressing cell growth in several cancers (NSCLC, hepatocarcinoma, skin and ovarian cancer) cell lines [21]. Oridonin from *Rabdosia rubescens* acts as an ATP competitive inhibitor of AKT1 and AKT2 and suppresses proliferation of esophageal squamous cell carcinoma in cell lines and in patient-derived xenograft tumors [8]. In silico models indicate that the flavonol herbacetin, found in ramose scouring rush herb and flaxseed, behaves as a dual inhibitor of ornithine decarboxylase (ODC) and AKT1/2 forming hydrogen bonds with the latter into the ATP binding pockets. Due to this interaction, tumor growth was suppressed in squamous cell carcinoma and melanoma [22]. However, to our knowledge, no clinical trials have been designed or are ongoing to assess the efficacy of natural inhibitors of AKT that directly target this kinase.

Based on this evidence and considering that the inhibition of PI_3_K/AKT pathway is associated with adverse events such as hyperglycemia and hyperinsulinemia [11], great interest is focused on the identification of new and specific inhibitors of the three AKT isoforms in different types of cancer.

The use of computational and bioinformatic approaches allows fast screening of molecules in the search for enzyme inhibitors and protein ligands. Starting by the experimental structure of a protein–ligand complex, it is possible to investigate by computational tools the structural features that other potential ligands should have, in order to be able to bind the protein. This procedure is known as the creation of a pharmacophore model, and it is followed by virtual screening, i.e., the search of a large data set of molecules in order to find candidate ligands that fit with the pharmacophore model [23]. Further computational studies can verify how the ligands may interact with the protein, as in the case of docking simulations [24].

In this article, we described our study aimed at identifying and investigating new compounds that could be potential candidates “lead compounds” to inhibit/modulate the activity of AKT1 protein [25,26]. Pharmacophore modeling has been applied by setting up a computational strategy protocol based on the integrated use of online and local tools for lead candidates’ generation-optimization. Experimental tests for preliminary validation of the effect of selected compounds have been performed.

## 2. Results

### 2.1. Computational Analysis

**Figure 1:**
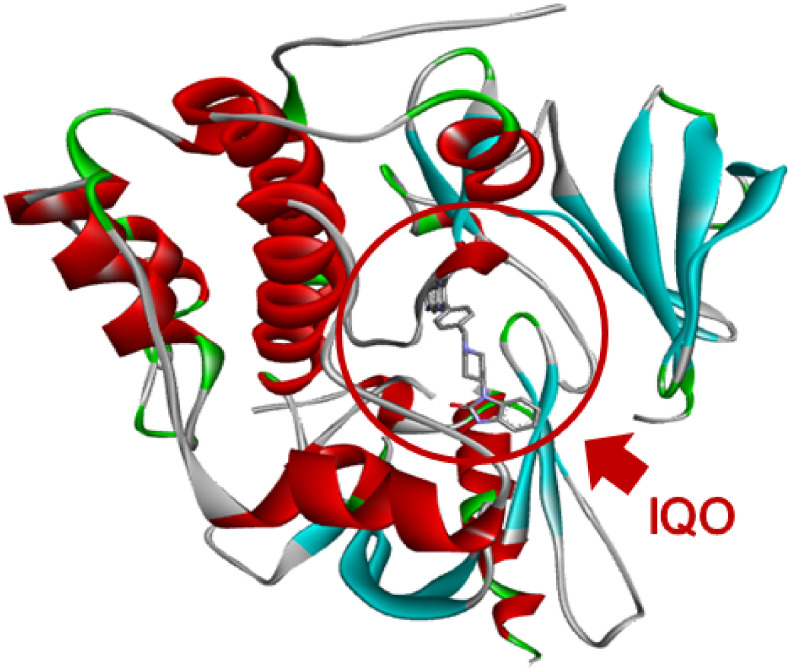
AKT1 structure with the IQO inhibitor. The image shows the global architecture of AKT1 and the IQO inhibitor position. The backbone of AKT1 is shown with red ribbons and cyan arrows to indicate helices and strands, respectively. The IQO molecule is represented in the stick mode, and its position is highlighted with the red circle.

**Figure 2:**
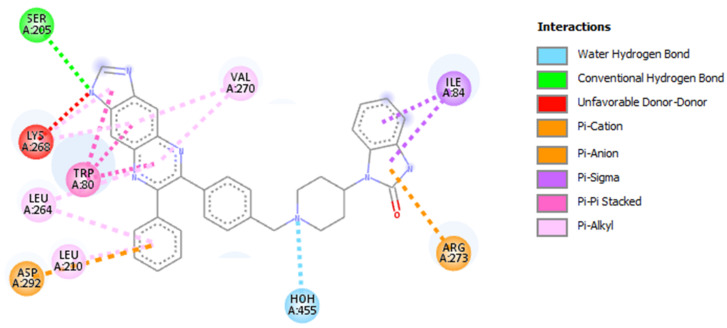
Amino acids of AKT1 that interact with IQO. The figure presents the schematization of the IQO interaction with AKT1, obtained with DiscoveryStudio. Van der Waals interactions are not shown to make more readable the scheme.

**Figure 3:**
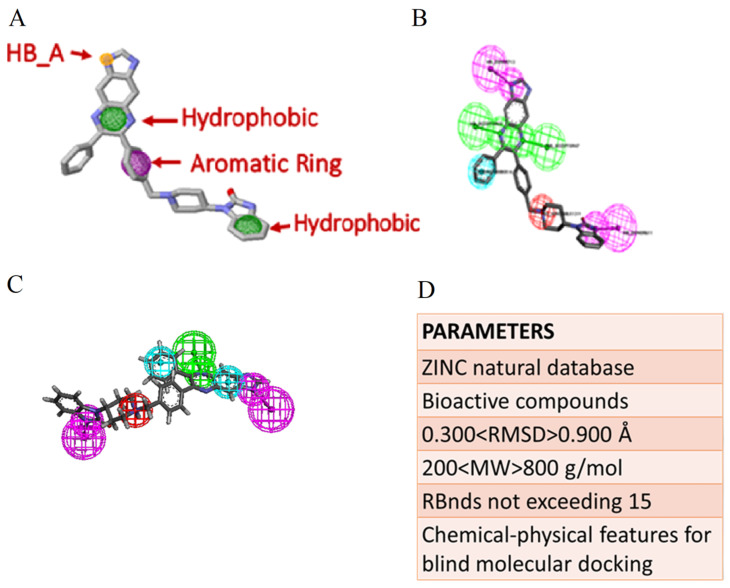
Pharmacophore models and data base screening. (**A**) The pharmacophore model built by Pharmit on the AKT1-IQO complex. (**B**) The pharmacophore model built by DiscoveryStudio on the AKT1-IQO complex. (**C**) The final pharmacophore model generated by DiscoveryStudio for the virtual screening. (**D**) The parameters used for the virtual screening.

To identify good candidates as potential drug-likes capable of modulating or inhibiting the activity of AKT1, the pharmacophore modeling strategy has been applied to the structure of AKT1 complexed with the inhibitor IQO (see Figure 1). The amino acids of AKT1 that interact with IQO have been analyzed with DiscoveryStudio (see Figure 2) and Ligplus (not shown). Both software identified Ser205 as involved in the H-bond interaction with IQO, and a number of amino acids involved in other interactions. Ligplus identifies them as generic hydrophobic interactions, whereas DiscoveryStudio gives more detailed description of their nature. These interactions are considered for the generation of the pharmacophore models based on the IQO interaction with AKT1. We analyzed by Pharmit and DiscoveryStudio the structure of the AKT1-IQO complex to generate different pharmacophore models, suitable to bind in a stable manner within the IQO binding site. Based on the pharmacophore models obtained by Pharmit and DiscoveryStudio (see Figure 3A,B), the starting parameters of the minimal pharmacophore model is characterized by four features (i.e., one aromatic ring, two hydrophobic regions and one hydrogen-bond acceptor).

The best pharmacophore model obtained is characterized by six features (Figure 3C), i.e., one H-bond donor, two hydrophobic, one positive charge and two aromatic rings. The large-scale screening for natural compounds in the ZINC data base matching the pharmacophore model (Figure 3D) generated a list of molecules (not shown), and the best candidates were further investigated by molecular docking simulations. Table 1 reports the best docking results obtained, together with the results deriving from docking the IQO molecule (redocking procedure) as a positive control, being a known inhibitor of AKT1, and quercetin (negative control) that is a specific ligand for PI*_3_*K but not AKT1 [20]. The known inhibitor of AKT1 interacts with a very low binding energy, i.e., <−12 Kcal/mol, while the selected compounds have binding energy values around −10 Kcal/mol, still suitable for a possible inhibitory effect. In the case of quercetin the energy value is higher, approximately −6 Kcal/mol, in agreement with the experimental evidence that quercetin is not a direct ligand of AKT1.

**Figure 4:**
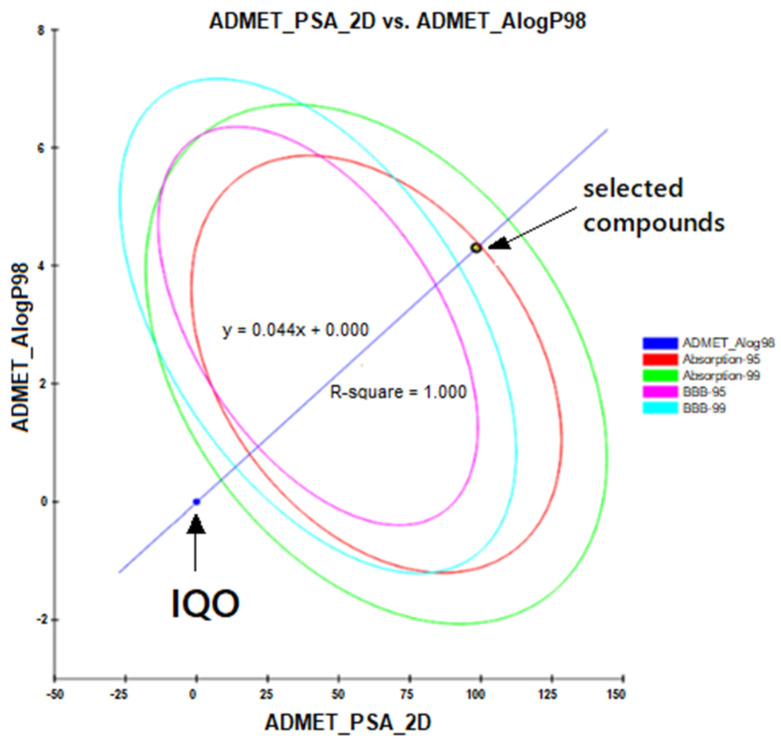
ADMET plot. The ADMET plot shows the relationship between the 2D polar surface area and the partition coefficient (n-octanol/water) algorithm, of the solution in which the bioavailability of the vitamin E compounds is calculated. The four ellipses define the regions where the inhibitors are expected to be well absorbed by the system and where they are expected to be located. In theory, for intestinal absorption, the inhibitors are well absorbed between 95 and 99% of the established confidence interval, if they fall within the ellipses colored in red and green, respectively. While, for absorption at the level of the blood–brain barrier, the inhibitors are well absorbed between 95 and 99% if they fall within the ellipses colored in magenta and cyan, respectively.

We performed a further analysis at PharmacoKinetics/PharmacoDynamics (PK/PD) level through the ADMET (Absorption, Distribution, Metabolism, Excretion, and Toxicity) predictor to study the bioavailability (adsorption, distribution, metabolism, excretion and toxicity) of the best selected compounds. Figure 4 reports the ADMET analysis for the best compounds selected by the virtual screening and molecular docking computational procedure. The analysis suggests that the compounds may be well absorbed by the intestinal barrier, not at level of the blood–brain barrier.

Then, we analyzed the toxicity profiles of the selected compounds by TOPKAT software integrated into Discovery Studio. Table 2 reports the results of the analysis. Different toxicity effects are predicted for the compounds, so we focused our attention on those without mutagenic or carcinogenic effects.

**Figure 5:**
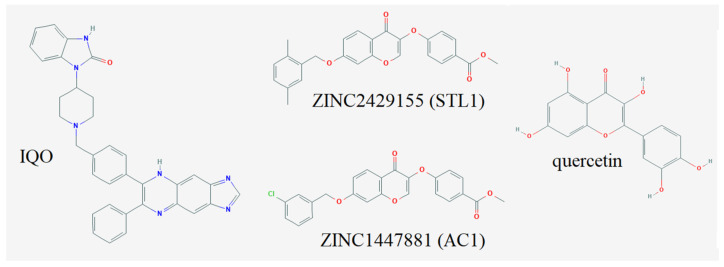
Chemical structures of the compounds investigated in the present study.

At the end of the computational study, the compounds we selected for the experimental validation were ZINC2429155 (also named STL1), ZINC1447881 (also named AC1) and ZINC02161363, having binding energy values in the range of −9/−10 Kcal/mol, Ki in the range of 1–2 μM, and favorable toxicity profiles. However, ZINC02161363 has not been tested for technical problems related to solubility. Figure 5 shows the molecular structure of the compounds selected for the experimental assays, together with IQO (also named iAKT-S), used as a positive control being a known inhibitor of AKT1. The structure of quercetin, used as a negative control, is also reported in Figure 5. 

### 2.2. Experimental Validation

To validate in a biological assay the results of the computational screening and predictions, we selected the cell line HG3 as a cellular model to assess in an in vitro model the inhibitory capacity of the putative AKT inhibitors on the activation of the kinase. 

**Figure 6:**
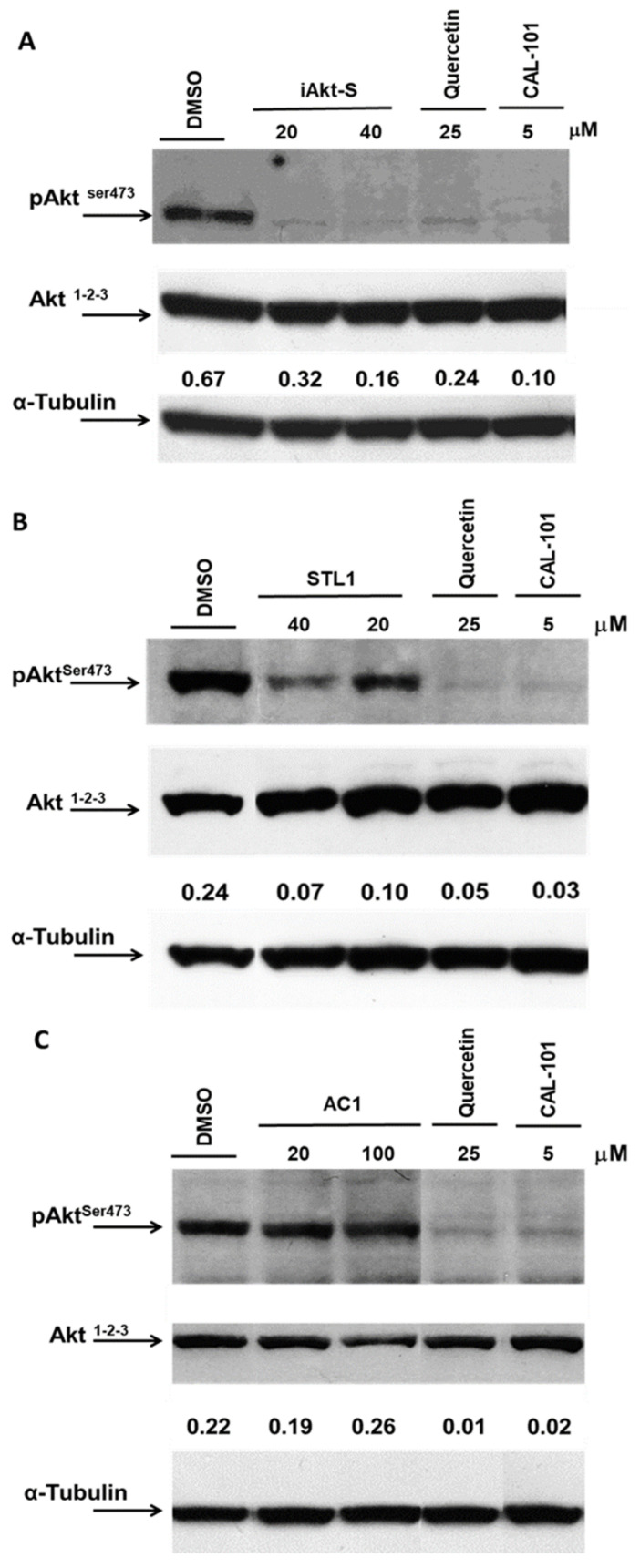
Effect of AKT inhibitors HG3 cells. Cells (1 × 10^6^/mL) were treated for 1 h with DMSO (0.1% *v/v*), quercetin (25 μM), CAL-101 (5 μM) and iAKT-S (IQO, 20–40 μM) (**A**); ZINC2429155 (STL1, 20–40 μM) (**B**) and ZINC1447881 (AC1, 20–100 μM) (**C**). Immunoblots were performed using the anti-phospho-AKT (p-AKT) antibody (upper panel). After two rounds of stripping, the blot was reprobed using the anti-AKT1-2-3 antibody (middle panel) followed by the anti-α−tubulin antibody. Densitometric analysis was calculated measuring optical density of p-AKT bands normalized respect to the expression of AKT and α-tubulin (pAKT/AKT/α-tubulin; numbers between the middle and lower panels). The blots are representative images of three independent experiments.

We firstly demonstrated that iAKT-S (IQO), employed as a positive control, was able to fully inhibit the phosphorylating activation of AKT on Ser473 (Figure 6A) and this effect was not due to changes in the AKT protein expression, which remained unchanged (Figure 6A, middle panel). Subsequently, we tested the compounds selected by the computational procedure. Surprisingly, only one of the two was able to inhibit AKT activation in HG3 cells. In fact, as reported in Figure 6B, STL1 dose-dependently inhibited pAKT-Ser473 expression of about 70%, based on densitometric analysis, at the highest concentration applied (40 μM). On the opposite, AC1, even at 100 μM concentration, did not change the phosphorylation state of pAKT-Ser473 compared to vehicle-treated (DMSO 0.1% *v/v*) cells (Figure 6C).

## 3. Discussion

Pharmacophore models make it possible to perform a large-scale screening of compounds applying several filters, to find good candidates to bind a target protein. A “good” candidate should be able to map all the parameters and all the chemical–physical characteristics required to carry out its biological activity. The compounds that match a well-defined pharmacophore have been analyzed through direct focused molecular docking for selecting only the best candidates and studying the protein–ligand interactions, taking into account the parameters related to the lowest binding energy and the estimated inhibition constant (Ki). This allowed us to evaluate which is the most stable complex and which is the compound that used at lower concentration is able to give the same biological results as the compounds used at higher concentrations.

To verify the AKT-inhibitory activity of the selected compounds, we selected the cell line HG3 for two main reasons. Firstly, previous studies from our laboratory demonstrated that two negative controls, quercetin, a natural compound, and CAL-101, a clinically relevant drug, were able to inhibit the PI_3_K/AKT pathway without directly binding AKT, but interacting with its upstream positive regulator PI_3_K [19,20]. Secondly, HG3 cells are resistant to the treatment of drugs able to inhibit antiapoptotic Bcl-2 family members, such as ABT-737, due to the overexpression of Mcl-1, a downstream effector of activating AKT in CLL and other leukemia [20,27]. Therefore, the identification of a new AKT inhibitor can lead to bypassing the drug resistance. 

IQO/iAKT-S is an allosteric AKT1/2 inhibitor [28] that, accordingly to Wu et al. [29], binds to the PH domain sequestering the kinase into the cytoplasm. In such a way, AKT cannot be recruited to the plasma membrane via interactions with the products of PI_3_K, be phosphorylated and activated on the two activation sites, Thr308 and Ser473. This is in agreement with our data shown in Figure 6A,B, where the phosphorylated and enzymatically active form of AKT, i.e., p-AKT-Ser473, is totally absent (IQO/i-AKT-S) or strongly reduced (STL1).

More complex is the interpretation of data referring to AC1. The easiest interpretation is that AC1 does not behave as an allosteric inhibitor of AKT; therefore, upon its addition to HG3 cells, AKT can move on the plasma membrane and be activated by mTORC2 phosphorylation on residue Ser473. This explanation conflicts with data in Table 1 and can be explained by evoking a reduced cellular uptake and a faster metabolism of AC1 compared to STL1, both limiting its intracellular concentration and capacity to bind to the AKT PH-domain. However, other explanations are plausible and may deserve further investigation. The assay reported in Figure 6 measures the “activation” of AKT, not its enzymatic activity. Being AC1 an allosteric inhibitor that does not compete with the ATP-binding pocket, we cannot exclude that the interaction between AC1 and the AKT PH-domain exists, but the conformational change does not “close” the AKT structure hiding the Ser473, as it happens for IQO/iAKT-S [29]. As a consequence, mTORC2 can still phosphorylate AKT, as shown in Figure 6C, which remains enzymatically inactive because of its binding to AC1. This hypothesis can be verified by designing an appropriate in vitro assay to measure the activity of the kinase and/or assessing the regulation of downstream effectors of the PI_3_K/AKT pathway to verify whether the treatment with AC1 modifies their status. Unfortunately, this latter solution cannot be easily pursued. In fact, for both STL1 and AC1, we demonstrated that the treatment did not significantly reduce HG3 cell viability, as we could expect for AKT inhibitors. The low capacity of both inhibitors to block cell proliferation can be explained considering the resistance of HG3 to proapoptotic and antiproliferative stimuli. Alternatively, it is necessary to keep in mind the limits common to several AKT inhibitors due to the high biochemical redundancy of the PI_3_K/AKT pathway associated with phenomena of its reactivation to bypass AKT inhibition (see Introduction). To this aim, we are currently investigating the possibility that the AKT inhibitors identified in the present study can show efficacy as anticancer agents in combination with other synthetic or natural compounds.

In conclusion, our computational strategy has been very effective in selecting a good “lead compound” as a potential inhibitor of AKT1 and PI_3_K/AKT1 pathway, as the experimental validation confirmed, and further compounds from our study could be an object of further investigation.

## 4. Materials and Methods

### 4.1. Reagents

Roswell Park Medium Institute (RPMI) medium, PBS, L-glutamine 200 mM, penicillin 5000 IU/mL/streptomycin 5000 μg/mL and 10% fetal bovine serum was from GIBCO ThermoFisher Scientific, Milan, Italy. Trypan blue solution, quercetin and dimetylsulfoxide (DMSO) were from Sigma-Aldrich (Milan, Italy). Cal-101 (GS1101) was purchased from Sellechem (Aurogene, Rome, Italy), divided into aliquots in DMSO and stored at −20 °C. IQO (iAKT-S), ZINC2429155 (STL1) and ZINC 1447881 (AC1) selected after in silico screening and dissolved in DMSO, were purchased from Vitas-M Limited, Hong Kong (https://vitasmlab.biz).

### 4.2. Cell Culture and Treatment

HG3 cells [30], a lymphoblastoid cell line with B1 cell characteristics established from a CLL clone by in vitro EBV infection, were cultured in RPMI medium supplemented with 10%, fetal bovine serum, 1% L-glutamine and 1% penicillin/streptomycin at 37 °C in a humidified atmosphere containing 5% CO_2_. Before starting each experiment, cells were counted with the Trypan blue system performed using the EVE Automatic cell counter (NanoEntek distributed by VWR, Milan, Italy) to assess their viability before starting each experiment (usually >90%) and expressed as the number of cells/mL.

The effect of AKT inhibitors on HG3 cells was tested treating 1×10^6^/mL cells for 1 h with DMSO, quercetin (25 μM), CAL-101 (5 μM), iAKT-S (IQO, 20–40 μM); ZINC2429155 (STL1, 20–40 μM) and ZINC1447881 (AC1, 20–100 μM). At the end of the incubation, the immunoblots were performed as reported below. In all untreated control experiments, we used the vehicle, DMSO, at a final concentration of 0.1% (*v/v*), which, from previous studies [19,20] resulted in not being cytotoxic for HG3 cells. Equal volumes of solutions (inhibitors and DMSO) were added to the cells.

### 4.3. Immunoblotting

After treatments with the different agents, HG3 cells were washed in PBS and suspended in lysis buffer containing 50 mM Tris/HCl; 150 mM NaCl; 1% NP-40; 10 mM EDTA; 10% glycerol; 0.5 mM DTT; 1% protease and phosphatase inhibitor cocktail (Sigma-Aldrich) and 100 μg of PMSF. Following measurement of protein concentration by the Bradford method [31], samples (30 μg) were added with Reducing Agent 20X (Bio-Rad Laboratories; Milan, Italy) and Sample Buffer 4× (TermoFischer-Scientific, Milan, Italy) consisting of: 100 nM Tris/HCL pH 6.8; 4% SDS; 200 nM of DTT; 20% glycerol and 0.2% bromophenol blue. Samples were boiled for 5 min and loaded on 4–12% polyacrylamide precast gel (Invitrogen Thermo Fisher Scientific). Electrophoresis was performed in the MOPS buffer ((3-(N-morpholino) propanosulfonic)) (50 mM MOPS, 50 mM Tris, 1% SDS, 1 mM EDTA; pH 7), at constant voltage (200 V) in an Invitrogen electrophoretic chamber. Proteins were transferred to a PVDF (polyvinyldenfluoride) membrane using the TRANS-Blot TURBO System (Bio-Rad Laboratories), with a constant amperage (2.5 mA) at room temperature. PVDF membranes were washed with T-TBS (0.1% Tween-20; 25 mM Tris; 137 mM NaCl and 2.69 mM KCl, pH 8) blocked 1 h at room temperature with 5% (*w/v*) non-fat dry milk in T-TBS before incubation with specific antibodies. The primary antibodies were: anti-pAKT-Ser473 (Cell Signalling, Milan, Italy; cat. #9271), anti-AKT1/2/3 (Santa-Cruz Biotechnologies distributed by DBA, Milan, Italy; cat. #sc-81434) and anti-α-tubulin (Sigma-Aldrich; cat. #T5168). PVDF membranes were finally incubated with horseradish peroxidase linked secondary antibody antimouse or rabbit IgG (GE Healthcare, Milano, Italy) and immunoblots revealed with the ECL Plus Western Blotting Detection System Kit (Perkin-Elmer, Milano, Italy). Band intensities were quantified measuring optical density on Gel Doc 2000 Apparatus and Multi-Analyst Software (Bio-Rad Laboratories).

### 4.4. Computational Procedure 

The bioinformatics/computational workflow is an advancement of the procedure applied in previous recent studies [32,33], and it is described in detail by the following steps.

(a) Selection of the structural model of AKT1. Search for a suitable structural model of AKT1 has been performed in PDB Protein Data Bank [34], looking for structures of AKT1 complexed with an inhibitor [35]. Among the available structures, by excluding those with mutations and limited portions of the protein, the most suitable resulted the PDB file with code 3O96, as the model crystal structure characterized by AKT1 protein linked to the interface to its selective allosteric inhibitor known as IQO (Inhibitor VIII of AKT1/2) [29]. Visual analysis of the 3D structure has been performed by means of Discovery Studio 4.5 (Biovia, San Diego, CA, USA). Analysis of the amino acids interacting with IQO has been performed by DiscoveryStudio and Ligplus [36].
(b)–(c) Pharmacophore modeling and virtual computational screening; the pharmacophore models have been obtained by integrating PHARMIT (http://pharmit.csb.pitt.edu) [37] and Discovery Studio 4.5 tools. ZINC12 database [38] has been searched for compounds of natural and bioactive origin and using Pharmer [39]. The molecular weight has been restricted to the range from 200 to 800 g/mol, RMSD to the range from 0.300 to 0.900 Å, RBnds (rotational angle) not exceeding 15.

The other parameters used to generate the 3D pharmacophore with PHARMIT and Discovery Studio, to apply “the Receptor-Ligand pharmacophore generation method” and “Best/Fast rigid conformation research method” are the following:

- Functional groups on which to implement pharmacophoric models (bonds that act as hydrogen acceptors/donors, bonds for positive/negative ions, hydrophobic bonds and presence of aromatic rings). A maximum of 10 hypotheses (or pharmacophore models) are generated for each run. The HypoGen algorithm used develops models with different pharmacophore features. The hypotheses generated are analyzed in terms of their correlation coefficients and the cost function values.
- Application of automatic parameter minimization based on Best/Fast rigid conformation characteristics. So, the FAST conformation generation method searches conformations only in the torsion space and takes less time. While, the BEST method provides a complete and improved coverage of conformational space by performing a rigorous energy minimization and optimizing the conformations in both torsional and Cartesian space using the Poling algorithm to assess the quality of pharmacophore hypotheses.
- Chemical–physical characteristics of the compounds were selected to perform the subsequent steps of work.
- Validation of the pharmacophore model: the pharmacophore models selected based on the acceptable correlation coefficient (R) and cost analysis should be validated in three subsequent steps: Fischer’s randomization test, test set prediction and the Güner–Henry (GH) scoring method. The method involves evaluation of the following: the percent yield of actives in a database (%Y, recall), the percent ratio of actives in the hit list (%A, precision), the enrichment factor E and the GH score. The GH score ranges from 0 to 1, where a value of 1 signifies the ideal model [40].

(d) After selecting a number of good candidates, blind and focused-rigid direct molecular docking has been performed by using AutoDock tools 4.2 [41], evaluating all the molecular interactions that may exist between the ligand and our AKT1 protein, taking into account the lower binding energy values to identify the most stable complex, and the estimated inhibition constant (Ki), to estimate which compounds are able to inhibit more AKT1 at lower experimental concentrations. Docking procedure has been applied according to protocols in use in our laboratory and described in previous articles [42,43,44]. Redocking procedure has been applied to evaluate the binding energy value of the IQO inhibitor as described in previous studies of our group [44].

The selected good candidates were analyzed using Discovery Studio to further refine the screening procedure, taking into account other parameters:

- The number of pharmacophore features must be included into the range between 4–6, taking into account that no pharmacophore has more than six functional groups present at the same time [45]). A pharmacophore model consisting of too many chemical features (e.g., more than six or seven) is not appropriate for practical applications. Therefore, it is always important to pick a restricted number of chemical features (usually four to seven) to create a reliable pharmacophore hypothesis. One more significant drawback is that the obtained pharmacophore hypothesis cannot replicate the quantitative structure–activity relationship (QSAR) because the model is generated based just on a single macromolecule–ligand complex or a single macromolecule.
- The selectivity score is a selectivity parameter of a ligand for a specific target to evaluate the quality of the pharmacophore models. There is no maximum limit (the higher the better).
- The method of generating the conformation of pharmacophores (FAST/BEST because they give us in a short time what are considered to be the best pharmacophores for the applied method) is used to then perform other internal screening and select only good candidates. The choice of these parameters is essential, to designate the subsequent steps of biological-molecular evaluation, taking only the best ones from the pharmacophores.

(e) The selected best candidates are then analyzed on a chemical–physical level using: Chemical vendors in Pubchem Compound [46] to identify the specifications of each single compound; SciFinder (scifinder.cas.org) to evaluate the presence or absence of preliminary tests already conducted on these compounds and what is already known in the literature on such compounds; FooDB/HMDB [47] to investigate food properties if there are ones and the origins of such chemical compounds and Chemicalize (chemaxon.com) to calculate the most important chemical parameters related to the stability of the compound. Afterwards, all the information collected were used to trace the origin of the compounds analyzed, underlining the most important features such as:
  - Solubility in organic solvents such as DMSO, ethanol, methanol and in inorganic solvents such as water;
  - LogD, pKa and the chemical stability of compounds.

(f) Molecular–biological evaluation of the selected compounds with the realization of the pharmacokinetic/pharmacodynamics (PK/PD) models. To select the “hit” compounds by virtual screening, it has been necessary to understand the features of pocket involved in ligand–protein interactions and to underline the amino acids involved in molecular interactions, trying to identify the compounds that can be considered good lead compounds. This step plays a critical role for the choice of the best candidates to calculate the physical–chemical properties and create PK/PD models, for characterizing the good lead compounds for next experimental assays.
(g) All this information are essential to realize the ADMET profile through ADMET/Toxicity predictor server (implemented in Discovery Studio), allowing the application of the Lipinski-Veber five rule, on which the bioavailability and the specific ADMET profile of each individual compound is calculated, and applying the TOPKAT software (implemented in Discovery Studio), useful for identifying and evaluating the toxicity profile of each compound in different conditions and systems.

## Figures and Tables

**Table 1:** Results of molecular docking simulations. The IQO compound is a known inhibitor of AKT1 and has been used as a positive control. Quercetin does not interact with AKT1 and has been employed as a negative control.

Compounds	Binding Energy (Kcal/mol)	Ki
IQO (iAKT-S)	−12.56	635.40 pM
ZINC4259855	−11.00	737.43 µM
ZINC2161363	−10.11	1.41 µM
ZINC2429155 (STL1)	−10.00	2.21 µM
ZINC1237912	−10.00	1.12 µM
ZINC1447881 (AC1)	−9.36	1.36 µM
ZINC13691379	−9.30	1.13 µM
ZINC02154548	−9.16	1.04 µM
ZINC03851635	−9.00	99.14 µM
ZINC02666313	−8.91	1.60 µM
ZINC14611917	−8.73	2.16 µM
ZINC54307082	−8.72	2.20 µM
Quercetin	−6.55	15.85 µM

Underline: these two compounds are those further investigated in the manuscript.

**Table 2:** Toxicity profiles of the selected compounds.

	Biodegradability	Test Ames: mutagen	Toxicity	FDA Mouse Female: Carginogen	FDA Mouse Male: Carginogen	RAT Female FDA: Carcinogen	RAT Male FDA: Carcinogen	Skin Irritancy	Skin Sensitizer	Predicted LD50 (mg/Kg)	Prediction Accuracy (%)
Compound											
**ZINC4259855**	no	no	no	no	yes	no	yes	mild	yes	100	67.4
**ZINC2161363**	yes	no	no	no	no	no	no	m/s ^a^	yes	100	67.4
**ZINC2429155**	no	no	yes	no	no	no	no	mild	yes	500	68.1
**ZINC1237912**	yes	no	no	no	no	no	no	m/s ^a^	yes	4400	54.3
**ZINC1447881**	no	no	no	no	no	no	no	mild	yes	500	68.1
**ZINC13691379**	no	no	yes	no	yes	yes	no	mild	yes	1000	67.4
**ZINC02154548**	yes	no	no	no	yes	yes	no	m/s ^a^	yes	600	54.3
**ZINC3869685**	no	yes	no	no	no	no	no	mild	yes	159	100.0
**ZINC02666313**	no	no	yes	no	no	no	no	mild	yes	100	67.4
**ZINC14611917**	yes	no	no	no	yes	yes	yes	m/s ^a^	yes	500	68.1
**ZINC54307082**	no	no	yes	no	no	no	no	m/s ^a^	yes	2875	68.1

^a^ m/s = moderate-severe; Underline: these two compounds are those further investigated in the manuscript.
